# Factors associated with time provided to children for physical activity in family child care: a cross-sectional study

**DOI:** 10.1186/s40064-016-3450-4

**Published:** 2016-10-06

**Authors:** Roger Figueroa, Angela Wiley

**Affiliations:** 1 Department of Human Development and Family Studies, University of Illinois at Urbana-Champaign, 230 Bevier Hall, M/C 180, 905 S. Goodwin Avenue, Urbana, IL 61801 USA; 2 Department of Human Development and Family Studies, University of Illinois at Urbana-Champaign, 2004 Christopher Hall, M/C 081, 904 W. Nevada Avenue, Urbana, IL 61801 USA

## Abstract

Childhood obesity has increased in the past 30 years, and physical inactivity is a major contributor. Factors related to physical activity promotion in the family child care context are understudied. A convenience sample of participants in a mid-sized city in the Midwestern U.S. was recruited through the local child care resource and referral agency and were invited through flyers and emails to take part in an online or paper survey. Survey results in a sample of 107 family child care providers indicate that many did not meet physical activity recommendations and are missing the opportunity to enable children’s physical activity via important practices and resources. Provider self-efficacy about being physically active, and indoor physical activity space positively associated with time provided for child physical activity. Health training is negatively associated with time provided for child physical activity. Practice implications include: (1) develop activities that promote physical activity in the tight confines of family child care homes and yard; (2) develop trainings that can influence the integration of suitable portable play equipment in the space constraints of family child care homes (3) Propose creative ideas for active free play even when in a shared space; (4) prioritize providing separate play areas by age group and strategize ways to do this in family child care contexts (for example, alternate access to spaces by age); (5) engage providers and children in joint activities that increase provider physical activity efficacy and physical activity time as well as that of children; (6) promote health and physical activity among family child care providers themselves.

## Background

Childhood obesity in the past 30 years has been on an upward trajectory even among our youngest with 10.4 % of U.S. preschool children classified as obese (Ogden and Carroll 2012). Given that childhood obesity is associated with poor health indicators well into adulthood (Singh et al. 2008), obesity prevention efforts must begin early. Strategic targets for such initiatives may include common environments where children spend a significant amount of time each day including child care (Story et al. 2006). More than 12 million preschoolers spend on average 33 h per week in early care and education settings (U.S. Census Bureau 2013), with multiple meals, snacks, and opportunities to engage in a mixture of physically active or sedentary behaviors. Of particular interest for this paper, physical activity plays a critical role in preventing obesity and promoting social and psychological development (Timmons et al. 2007, 2012). Given that time spent in child care environments is an important predictor of physical activity for young children (Finn et al. 2002; Pate et al. 2004), there is a clear need to better understand features of early care and education settings and providers that may promote or hinder children’s physical activity.

While young children need between 2 and 3 h of structured and unstructured physical activity each day (National Association for Sport and Physical Education 2002, 2009; Pate and O’Neill 2012) or more than 15 min per hour of care (Pate et al. 2015), most preschoolers fail to meet this guideline (Beets et al. 2011; Delaney et al. 2014; Pate et al. 2015). Evidence suggests that there is notable variation in the amount of preschoolers’s physical activity (Pate et al. 2004) with many engaging in less than half of the recommended minimum 15 min per hour of care. In one recent study, less than half of preschoolers were physically active for an average of 15 min per hour in care (Pate et al. 2015) and another documented only 9 min per hour (Delaney et al. 2014). In order to meet existing guidelines, the child care environment is critical to promote physical activity participation among preschoolers attending these settings (Cosco 2006; Finn et al. 2002). Among the multiple factors that contribute to low levels of preschoolers’ physical activity in child care settings, providers’ physical activity practices and organization of the environment are known correlates that require further examination (Delaney et al. 2014; Tandon et al. 2016).

There are two types of child care, namely center-based care and home-based care. Center-based care typically involves multiple providers and often age-segregated classrooms in a common public facility, whereas home-based care (or family child care) most often occurs in private homes with smaller groups of mixed-age children (OPM 2016). Family child care settings are the second largest provider of non-relative care in the U.S. for preschoolers (Forum on Child and Family Statistics 2009). This setting is an important context for promoting health (Kim et al. 2011) and encouraging preschoolers’ physical activity (Delaney et al. 2014; Tandon et al. 2016). Though many children are in family child care at any given time, these sites remain the least researched of child care types perhaps because these home-based businesses are logistically challenging to include in studies (Gunter et al. 2012).

Among practices that can impact preschoolers’ physical activity, one of the most basic practices is time provided for children to be physically active as a routine in providers’ daily care program (Finn et al. 2002). Without time to be active, children are unlikely to engage in physical activity. In the context of daily programming in child care centers, evidence suggests that time provided for children to be physically active varies depending on environmental factors such as available indoor and outdoor space, and play equipment (Mulligan et al. 1998; Finn et al. 2002). Gunter et al. (2012) found that children engaged in more minutes of physical activity in family child care programs that set time aside for daily outdoor active play; and had physical activity resources available such as portable play equipment, fixed play equipment, and adequate indoor play space (Gunter et al. 2012). However, little is known about how time provided for children to be physically active is related to the availability of indoor play spaces, portable play equipment, and other providers’ characteristics.

Among other physical environment characteristics, indoor play spaces and portable play equipment are especially important in promoting children’s physical activity in family child care settings (Fees et al. 2009; Gunter et al. 2012). Like other aspects of family child care environments these are likely to vary widely, and we need to better understand how variations in these factors are related to variations in children’s physical activity patterns in family child care settings (Cosco 2006).

Health-related training may also influence family child care providers’ physical activity programming (Fees et al. 2009; Gunter et al. 2012). Kim et al. (2011) found that obesity related training is associated with center-based and home-based providers’ practices and perceptions about children’s health. Health-related training has also been conceptualized as a facilitating factor and foundation for physical activity practices in family child care (O’Connor and Temple 2005).

Self-efficacy is an important characteristic that influences parenting practices (Jones and Prinz 2005) and may well influence child care provider practices related to children’s physical activity. A concept first introduced by Bandura (1977), “self-efficacy” refers to the extent to which people feel capable of performing specific behaviors to attain certain goals. Self-efficacy to be physically active is a predictor of engaging and persisting in physical activity (McAuley and Blissmer 2000). It may be that self-efficacy to be physically active oneself and/or self-efficacy to impact children’s physical activity is related to time that family child care providers set aside for children’s physical activity.

This study will address gaps in our understanding of physical activity promotion within the family child care context while providing a research base for future training and efforts to optimize physical activity in this setting. The specific objectives of this study are to (1) describe relevant family child care physical activity environment, (2) and to explore how providers’ (a) available indoor space and portable equipment resources, (b) health training, (c) physical activity self-efficacy, are related to time provided to children for physical activity.

## Methods

### Procedures

As part of a larger project, the Family Child Care Health Study began data collection procedures November 2013 in a mid-sized U.S. Midwestern city. This cross-sectional pilot survey included multiple validated assessments of family child care providers’ demographic characteristics (e.g., age, gender, household income, level of education), health, health-related behaviors, and health-related practices. Participants could choose to complete the survey either online or as a paper copy sent and returned in the U.S. mail.

### Participants

A convenience sample of participants was recruited through the local child care resource and referral agency and licensed family child care providers were invited through flyers and emails to take part in an online or paper survey. Interested licensed family child care providers were screened for eligibility (e.g., they had to be licensed family child care providers and not center providers) and informed consent was obtained according to a protocol approved by the Institutional Review Board (IRB) at the sponsoring university. A total of 107 licensed family child care providers (all female) participated in this study.

### Measures

#### Time provided for physical activity

Time provided for children to be physically active serves as the dependent variable. It was assessed using an item from the Infant and Child physical activity version of the Go NAP SACC Physical Activity Self-Assessment (Ward et al. 2014): “The amount of time provided to preschool children for indoor and outdoor physical activity each day.” The response scale was (1) “Less than 60 min (Half-day: Less than 30 min)” (2) “60–89 min (Half-day: 30–44 min)” (3) “90–119 min (Half-day: 45–59 min)” (4) “120 min or more (Half-day: 60 min or more).”

#### Physical activity equipment and indoor play spaces

Physical activity equipment and indoor play spaces were assessed using survey items from the Go NAP SACC Physical Activity Self-Assessment. To learn about their indoor play space, participants were asked: “My program offers how many of the following indoor play space features: (1) space for all activities, including jumping, running, and rolling; (2) separate play areas for each age group; (3) areas that allow play for individuals, pairs, small groups, and large groups; (4) space that is fully access for children with special needs”. The number of features in each subcategory was summed to give a total “indoor play space” score ranging from 0 to 14.

 To learn about their equipment, participants were asked: “My program has the following portable play equipment features available in good condition for children to use indoors (indicate how many of each):” (1) jumping toys, e.g., jump ropes, jumping balls; (2) push–pull toys, e.g., wagons, wheelbarrows, big dump trucks; (3) twirling toys, e.g., ribbons, scarves, batons, hula hoops, parachutes; (4) throwing, catching, and striking toys: balls, bean bags, noodles, rackets; (5) balance toys, e.g., balance beams, plastic “river stones”; (6) crawling or tumbling equipment, e.g., mats, portable tunnels. The number of features in each subcategory was summed to give a total “equipment” score ranging from 0 to 33. Alpha coefficients (Cronbach 1951) indicated acceptable internal consistency for space (0.723) and equipment (0.873) across all of their respective sub-categories.

*Health training* Family child care providers were asked if they have had health related training in the past year using a single item. Participants answered with a dichotomous yes or no response.

*Self-efficacy* Provider self-efficacy to be physically active themselves was measured using one item (Hayes 2010): “How confident are you in your ability to ensure that you get the recommended amount of moderate to vigorous physical activity?” The response scale was (1) “Not at all confident,” (2) “Somewhat confident,” (3) “Moderately confident,” (4) “Very confident,” (5) “Completely Confident.” In addition, we measured providers’ self-efficacy to influence children to be physically active by adapting Hayes (2010) item and using the same response scale: “How confident are you in your ability to ensure that the children in your care get the recommended amount of moderate to vigorous physical activity?”

*Other provider characteristics* A range of provider characteristics was gathered including age, race and ethnicity, marital status, income, education, weekly physical activity behavior, and perceived health. Age, race and ethnicity, and education were adopted from a survey of salary and staffing for family child care providers in 2011 (IDHS 2011). Marital status, income and self-rated health were survey items adapted from the Behavioral Risk Factor Surveillance Survey (CDC 2013). Weekly physical activity behavior was asked using items from the Global Physical Activity Questionnaire (WHO 2012).

### Statistical analyses

Descriptive statistics summarize provider characteristics and physical activity practices/resources. Bivariate correlations explored relationships among these. An Ordinary Least Square (OLS) regression model examined the predictive validity of provider characteristics and physical activity resources on physical activity time provided for children. Age, health-related training and household income were included in our OLS regression model as control variables, whereas other variables such as gender, ethnicity, education and play equipment were not included for methodological reasons (e.g., homogeneity in the sample and to avoid co-linearity). Lastly, effect sizes were calculated for the OLS regression model. According to Cohen (1992), an effect size 0.02 is considered small effect, 0.15 is considered a medium effect, and 0.35 is considered a large effect (reported in the result section). Statistical software STATA was used to perform all analyses.

### Multiple imputations analysis

Data gathered from the survey contained some missing values for different variables (including “do not know” responses) with no obvious patterns in the missing values across variables and participants. Listwise deletion of the data (keeping only participants with no missing values on any items) would have reduced our sample to approximately 65 % of the participants. Therefore, we assumed that the data were missing at random (MAR), and replaced the missing values using multiple imputation (Rubin 1976, 2004; Schafer et al. 1998). Ten complete datasets were created from the original using the multiple imputation (MI) command by the ICE/FCS imputation functionality package for STATA 12 and above (Bartlett 2012). Multivariate Imputation of theoretically relevant variables in the model accounted for missingness in the following variables: time provided for physical activity (12 %), provider self-efficacy (11 %), Self-efficacy to influence child physical activity (13 %), age (1 %), income (2 %), education (5 %), weekly physical activity (25 %) and self-rated health (8 %).

## Results

The majority of family child care providers in this sample were between the ages 30–59. Most were married (71 %) and approximately 91 % were Caucasian. Over half of the respondents (55 %) reported household incomes under $50,000 per year while over two-thirds of the sample had less than a college degree (78 %). The majority of participants rated their health as good, or better. Moderate physical activity was reported on average about 4 days per week. Early childhood related education was limited to bachelor’s degree or less. The majority of participants had health-related training and professional development related to physical activity at least once during their time as a provider (Table 1).

**Table 1:** Descriptive characteristics of the sample

	% of respondents
Age	
20–29 years	3
30–39 years	24
40–49 years	35
50–59 years	27
60 years +	11
Race	
African-American	7
Caucasian/white	91
Other	2
Marital status	
Married	71
Divorced	14
Widowed	5
Other	10
Income	
Less than $25 K	18
$25 K to <$35 K	14
$35 K to <$50 K	23
$50 K to <$75 K	22
$75 K or more	23
Education	
HS or less	34
Some college	31
Associates degree	13
Bachelor’s degree	22
Self-rated health	
Excellent	17
Very good	49
Good	28
Fair	6
Poor	0
Physical activity	
Avg. days in MPA	4.12
Health training	
Yes	56
No	44

Descriptively speaking, fewer than half of the respondents reported routinely providing children with guideline recommendations of 120 min of active playtime each day (47 %). While family child care providers are encouraged to lead physical activity routines for children twice per day, 32 % of our sample rarely or never set up physical activity routines. With regards to play space, indoor play space available for all activities is recommended, yet about 32 % of our sample does not provide separate play areas for different age groups (McWilliams et al. 2009). With respect to gross motor promoting play equipment, 52 % did not have any balance toys while 24 % did not have any balls/ropes/jumping toys. More than a quarter of providers (27 %) did not provide separate play areas by age group.

Table 2 presents the bivariate correlation matrix for relevant variables. There was a significant low correlation between physical activity space and time provided for physical activity, and between providers’ self-efficacy to influence child physical activity and time provided for physical activity. In addition, a significant moderate correlation was noted between provider self-efficacy to be active and time provided for physical activity. A negative low correlation was also found between health-related training and time provided for physical activity. There was no correlation between physical activity equipment and time provided for physical activity. Additionally, we found high colinearity between physical activity space and equipment. Physical activity equipment, self-rated health, providers’ weekly physical activity and education were removed from further analyses since no bivariate relationships were found with time provided for physical activity and to increase the efficiency of the regression model.

**Table 2:** Pair-wise Correlation Matrix between time provided for physical activity, physical activity space, physical activity equipment, self-efficacy, self-efficacy of influencing child physical activity, self-rated health, health training, active days/week, age, income, and education

	1	2	3	4	5	6	7	8	9	10	11
1. PA time provided	1.0000										
2. PA space	0.27***	1.0000									
3. PA equipment	0.16	0.60****	1.0000								
4. Self-efficacy	0.31***	0.37****	0.27***	1.0000							
5. Self-efficacy for child PA	0.23**	0.11	0.13	0.26***	1.0000						
6. Self-rated health	0.03	−0.08	−0.18*	−0.27***	−0.06	1.0000					
7. Health training	−0.24***	0.24***	0.27***	0.20**	0.03	−0.28***	1.000				
8. Active days/week	0.18	0.04	0.18*	0.40****	0.13	−0.02	0.15	1.000			
9. Age	−0.16	−0.05	0.00	−0.06	−0.07	−0.10	0.00	−0.02	1.000		
10. Income	−0.19*	−0.01	0.02	0.06	0.00	−0.20**	0.21**	0.00	−0.08	1.000	
11. Education	0.02	−0.01	0.03	0.02	0.30***	−0.02	0.14	0.16	−0.08	0.07	1.000

**** P < 0.001; *** P < 0.01; ** P < 0.05; * P < 0.10

An ordinary least squares (OLS) regression model examined the effects of provider self-efficacy to be physically active, provider self-efficacy to influence children to be active, physical activity space and health training on time provided for children’s physical activity (Table 3). Providers’ self-efficacy to be physically active is positively associated with time provided to time provided to children for physical activity (*P* < 0.01; effect size: 0.05), and physical activity space was positively associated with time provided for physical activity (*P* < 0.01; effect size: 0.04). Additionally, health training was negatively associated with time provided for physical activity (*P* < 0.001; effect size: 0.10). These relationships were found while accounting for age and household income.

**Table 3:** Ordinary Least Squares regression model predicting time provided for physical activity while accounting for physical activity space, self-efficacy, self-efficacy of influencing child physical activity, health training, age, and income

Variables	Coefficient (SE)
PA space	0.08 (0.03)***
Self-efficacy	0.23 (0.09)***
Self-efficacy of child PA	0.13 (0.10)
Health training	−0.70 (0.22)****
Age	−0.15 (0.09)*
Income	−0.09 (0.05)*
Constant	2.90 (0.71)****

**** P < 0.001; *** P < 0.01; ** P < 0.05; * P < 0.10

## Discussion

The purpose of this study was to describe family child care provider characteristics and those of the family child care physical activity environment, determine whether family child care providers and family child care physical activity environmental resources meet national guidelines for physical activity practices; and explore how providers’ indoor space and portable equipment resources, physical activity self-efficacy and health training, are related to time provided for child physical activity. It is worth noting that many family child care providers do not meet expert recommendations in a number of important areas for physical activity promotion in child care settings. This study contributes to the emerging literature that demonstrates a need for effective programs to promote physical activity in family child care (Temple et al. 2009; Gunter et al. 2012; Rice and Trost 2014).

This study sought to explore factors that may impact the provider practice of providing time for children’s physical activity in family child care. Family child care homes vary widely in their physical environment and are subject to little oversight and routinization imposed from a director, given that family child care providers are typically independent business owners. The characteristics and practices of the individual provider may matter even more in this context than in centers.

Space is clearly important for the physical activity of children in child care. Evidence suggests that children are more active when they have adequate space and are afforded time for physical activity (Bower et al. 2008; Gunter et al. 2012). In this study, we gave special attention to the availability of indoor play space as this has been identified in qualitative studies as a potential barrier to providing children with physical activity opportunities in family child care settings (e.g., Fees et al. 2009). In a key finding from our data, space resources do indeed impact the provider practice of setting aside time for children’s physical activity. Family child care providers typically have a work environment that overlaps with their personal home environment, meaning that these homes’ space is shared and often multifunctional. The spaces available for children’s play and physical activity are constrained by this reality, and limited space in a child care setting is known to restrict movement (Finn et al. 2002). Further attention needs to be given to the shared space in family child care settings to avoid jeopardizing the time afforded to children for physical activity (Squibb and King 1996). The current finding supports a growing body of work calling for further understanding of how to optimize family child care space and promote dedicated time for children’s physical activity in family child care settings.

No associations emerged between physical activity equipment and time provided to children for physical activity. Previous literature suggests that family child care providers often do not receive training about the arrangement and use of equipment/materials, at least not training tailored to the realities of their home-based business (Squibb and King 1996). While training is assumed to be a crucial foundation for health promotion in child care settings (Kim et al. 2011), general health-related training was negatively related to time provided for physical activity among this sample. It may be that more nuanced assessment of training would shed further light on this finding; it may also indicate that the content of health-related trainings is not adequately conducive of promoting physical activity in family child care settings. Better physical activity practices may be tightly linked to more tailored training.

Self-efficacy around physical activity was related to provider’s physical activity practices in this study. Providers’ self-efficacy to impact children’s physical activity was correlated with the time provided for physical activity. This is consistent with another study that found providers’ self-efficacy for impacting children’s healthy eating habits to be associated to their child feeding practices (Lanigan 2012). This association warrants further exploration given that family child care providers in particular may feel capable of impacting children’s health outcomes, perhaps due to the close and personal nature of the home-based setting (Kim et al. 2011). Providers’ self-efficacy to be physically active themselves was an important predictor of the time they provide to children for physical activity. A provider who is uncomfortable with physical activity herself may be reluctant to set aside time for children to be active while in her care, feeling unable to model for them or engage in joint activity. This suggests that interventions to improve physical activity-related provider practices should address the physical activity self-efficacy of providers in addition to environmental resources for physical activity.

## Conclusion

The availability of indoor space, providers’ self-efficacy to be physically active themselves, and their health-related training are important features associated to setting time aside for children to be active in family child care. This highlights the importance of enhancing these factors among family child care providers in order to better promote physical activity among young children in their care.

## Practice implications

Many family child care providers in this study were not meeting recommendations for promoting children’s physical activity. While some of these gaps may be related to the constraints of the home environment, tailored training can help family child care providers capitalize on opportunities to enable preschoolers’ physical activity by adapting their practice within the constraints of their physical space. Specific strategies include: (1) develop tailored activities that promote physical activity in the tight confines of family child care homes and yard; (2) develop trainings that can influence the integration of suitable portable play equipment in the space constraints of family child care homes (3) Propose creative ideas for active free play even when in a shared space; (4) prioritize providing separate play areas by age group and strategize ways to do this in family child care contexts (for example, alternate access to spaces by age); (5) engage providers and children in joint activities that increase provider and children’s physical activity self-efficacy and physical activity participation; and (6) promote health and physical activity among family child care providers themselves.

## Limitations

Studies of family child care are relatively rare in part given the logistical challenges of reaching to participants who are scattered across private homes, employed and most often without support to participate in research studies. The sample size in this pilot study is relatively small; thus, more participants and more complete data would increase our power to build nuanced statistical models. The Go NAPSACC-SA is considered a reliable and valid instrument commonly used in child care settings, however, the questions are general and the self-report data are limited. Additionally, although psychometric properties of this item are available elsewhere (Benjamin et al. 2007), this kind of assessment may need further development for the use by researchers. Another limitation in this study was not investigating the outdoor space and its relationship to the study outcomes. Outdoor play is an important area that warrants future exploration in these sites, as the build of the outdoor environment may influence how much time providers set aside for children’s physical activity. The cross-sectional nature of the current study limits interpretation of the data, as does the ethnic and racial homogeneity of the sample. Education and income were spread out almost evenly in our sample and were able to be accounted for in our OLS regression model. Nevertheless, future studies should explore these relationships with in more diverse populations while accounting for socio-demographic factors and theoretically relevant physical activity constructs.
